# Case Report: Regenerative hepatic pseudotumor induced by tislelizumab in a lung cancer patient

**DOI:** 10.3389/fimmu.2025.1565065

**Published:** 2025-05-30

**Authors:** Wenrui Wang, Wei Li, Tianqi Zhang, Zhenjing Jin, Lanlan Yang

**Affiliations:** ^1^ Digestive Diseases Center, Department of Hepatopancreatobiliary Medicine, The Second Hospital, Jilin University, Changchun, China; ^2^ Department of Respiratory and Critical Care Medicine, The Second Hospital of Jilin University, Changchun, Jilin, China; ^3^ Department of Radiology, The Second Hospital of Jilin University, Changchun, China

**Keywords:** immune checkpoint inhibitors, immune-related adverse events, non-small cell lung cancer, regenerative hepatic pseudotumor, tislelizumab, hepatic immune toxicity, PD-1 inhibitors, immunotherapy complications

## Abstract

Immune checkpoint inhibitors (ICIs) have revolutionized the treatment for different types of cancers, providing significant clinical benefits. However, these therapies are associated with various immune-related adverse events (irAEs), including hepatic manifestations such as hepatitis, sinusoidal obstruction syndrome (SOS), and nodular regenerative hyperplasia. Among these, regenerative hepatic pseudotumors (RHPs) are exceptionally rare and poorly described in literature. Here, we report the case of a 66-year-old man with metastatic non-small-cell lung cancer (NSCLC) who developed a hepatic pseudotumor during routine imaging following treatment with the anti-programmed cell death 1 (PD-1) therapy, tislelizumab. Despite the presence of a hepatic lesion on imaging, the patient exhibited no clinical symptoms or biochemical evidence of severe immune-mediated hepatitis. Following cessation of anti-PD-1 therapy and initiation of systemic steroid therapy, the hepatic pseudotumors stabilized without further growth. The findings suggest that ICI therapy may be associated with the development of regenerative hepatic pseudotumor (RHP). Given the nonspecific and potentially misleading imaging features of RHP, biopsy is essential for accurate diagnosis and differentiation from malignant lesions such as hepatic metastases. Early histological evaluation through biopsy can prevent unnecessary interventions and guide appropriate management in patients presenting with liver lesions during or after ICI therapy. This case suggests a possible association between the development of RHP and tislelizumab treatment. The effect of ICI-induced hepatic pseudotumors on NSCLC progression is unclear and requires further investigation.

## 1 Introduction

Immune checkpoint inhibitors (ICIs) are a class of immunotherapy drugs that enhance the immune system’s ability to recognize and destroy cancer cells (1). They target key regulatory pathways, including programmed cell death protein 1 (PD-1), programmed death-ligand 1 (PD-L1), and cytotoxic T-lymphocyte-associated protein 4 (CTLA-4), which are often exploited by tumors to evade immune surveillance (2). By blocking these inhibitory signals, ICIs restore T-cell activation and promote antitumor immunity.PD-1 inhibitors, such as tislelizumab, nivolumab, and pembrolizumab, specifically target the PD-1 receptor on T cells, preventing its interaction with PD-L1 expressed on tumor cells and immune-suppressive cells (3). This blockade enhances T-cell activity, leading to tumor cell destruction. PD-1 inhibitors have demonstrated significant efficacy in treating various malignancies, including melanoma, non-small cell lung cancer, and hepatocellular carcinoma (4). However, their use is associated with a unique spectrum of immune-related adverse events (irAEs) due to the activation of the immune system.irAEs are a diverse group of side effects resulting from the non-specific activation of the immune system by ICIs. They can affect virtually any organ system with the most common being:dermatologic (rash, pruritus, and vitiligo) (5), gastrointestinal(colitis, diarrhea, and hepatitis) (6), endocrine(hypothyroidism, hyperthyroidism, and hypophysitis (1)), hepatic(immune-mediated hepatitis, nodular regenerative hyperplasia, and regenerative hepatic pseudotumor (RHP) (6, 7)), pulmonary(pneumonitis, which can be life-threatening) (5), rheumatologic(arthritis and myositis) (1). The severity of irAEs varies from mild to life-threatening, often necessitating temporary or permanent discontinuation of ICIs and the use of immunosuppressive therapies such as corticosteroids (5). Among these, liver damage is a rare but well-known adverse event associated with anti-PD-1 antibodies (8). This damage encompasses a spectrum of manifestations, from asymptomatic liver enzyme elevation to immune-mediated hepatitis. Histopathological features in immune-related hepatitis include panlobular hepatitis, bile duct injury, portal phlebitis, granuloma formation, steatosis or steatohepatitis, nodular regenerative hyperplasia, and secondary sclerosing cholangitis (9).

Tislelizumab is a humanized monoclonal antibody that selectively targets the programmed cell death protein 1 (PD-1) receptor. Its unique design minimizes binding to Fcγ receptors on macrophages, thereby reducing antibody-dependent phagocytosis and enhancing antitumor activity. Tislelizumab has demonstrated significant clinical efficacy in the treatment of various malignancies, including non-small cell lung cancer, hepatocellular carcinoma, and classical Hodgkin lymphoma (10). As a PD-1 inhibitor, tislelizumab enhances T-cell-mediated immune responses, leading to improved antitumor effects. However, its use is also associated with a spectrum of immune-related adverse events (irAEs), which can affect multiple organ systems (6). Here, we report the case of a patient with advanced NSCLC who developed a regenerative hepatic pseudotumor following treatment with Tislelizumab. This has rarely been reported as an adverse effect of this therapy. This report underscores the importance of clinicians being aware of the presence of liver changes in patients undergoing ICI therapy. Biopsy should be considered, when necessary, to prevent misdiagnosis and inappropriate immunotherapy adjustments.

## 2 Case description

**Figure 1:**
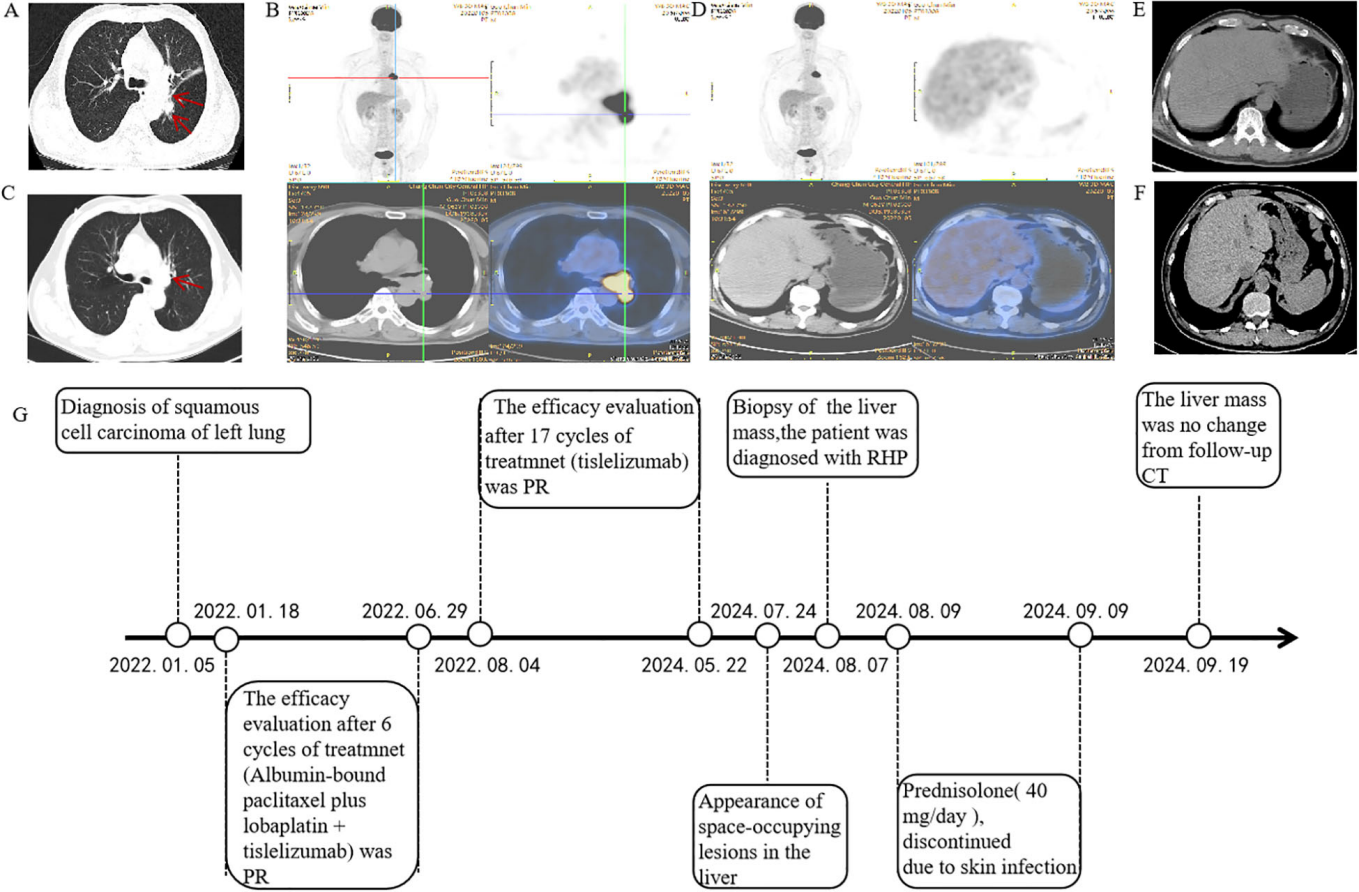
Positron emission tomography/computed tomography (PET/CT) and computed tomography (CT) of the thorax with contrast at the time of diagnosis. **(A)** Thoracic CT revealed multiple masses (superior lobe of the left lung and left hilar lymph nodes) (2022.01.05). **(B)** Positron emission tomography/computed tomography revealed a suspicious primary lung cancer and metastasis (2022.01.15). **(C)** A partial response was achieved, resolving after six cycles (2022.06.28). **(D, E)** Prior to treatment, a PET scan showed no hepatic lesions were identified (2022.01.05). **(F)** CT showed normal liver morphology during treatment (2023.03.08). **(G)** Timeline of disease and treatment.

A 66-year-old Asian male patient presented to the Department of Respiratory and Critical Care Medicine at the Second Hospital of Jilin University in December 2021, with complaints of cough and sputum. The patient presented with a 2-month history of cough with expectoration and a 5-day history of breathlessness. Physical examination findings were unremarkable.Computed tomography (CT) of the chest revealed peripheral lung cancer in the left inferior dorsal region with hilar lymph node metastasis (**Figure 1A**). Subsequent sodium fluoride (18F-NaF) positron emission tomography/computed tomography (PET/CT) confirmed the lesions identified on CT (**Figure 1B**). Tumor marker analysis showed carcinoembryonic antigen (CEA) levels of 10.74 ng/mL (normal range 0–5 ng/mL) and cytokeratin 19 fragment (CYFRA 21-1) levels of 4.04 ng/mL (normal range 0–2.08ng/mL). Fiberoptic bronchoscopic biopsy revealed NSCLC in the lower left main airway, with morphology consistent with moderately differentiated squamous cell carcinoma. Laboratory tests showed normal liver function parameters: aspartate aminotransferase (AST) (20 U/L; normal range: 15–40 U/L),alanine aminotransferase (ALT) (23 U/L; normal range: 9–50 U/L), gamma-glutamyl transpeptidase (GGT) (40 U/L; normal range: 10–60 U/L), alkaline phosphatase (AKP) (41 U/L; normal range: 45–125 U/L), and total bilirubin (3.58 μmol/L; normal range: 2.00–20.10 μmol/L). Pre-treatment imaging results of the liver are presented in (**Figures 1D, E**). His medical history included coronary stent implantation 14 years prior, and he was currently on regular aspirin therapy. He denied any history of hypertension, diabetes mellitus, asthma, liver disease, or other chronic conditions. Additionally, the patient had no history of smoking, alcohol consumption, or hereditary diseases, and his family medical history was unremarkable.

According to the 2022 National Comprehensive Cancer Network (NCCN) guidelines, systemic chemotherapy combined with immunotherapy is recommended as the first-line treatment option. The patient received six cycles of first-line chemotherapy with albumin-bound paclitaxel plus lobaplatin, combined with tislelizumab immunotherapy. Partial response was achieved and persisted after 6 cycles (**Figure 1C**). The patient exhibited no significant adverse event other than a slight decrease in white blood cell count. Subsequent abdominal CT imaging revealed no significant abnormalities (**Figure 1F**) .Throughout the treatment course, regular monitoring of routine blood tests, liver and kidney function, immunological indices (including cardiac enzymes, thyroid function, and pituitary function), electrocardiograms, abdominal imaging, and cardiac ultrasound revealed no significant abnormalities. The timeline of treatment course was summarized in **Figure 1G**.

**Figure 2:**
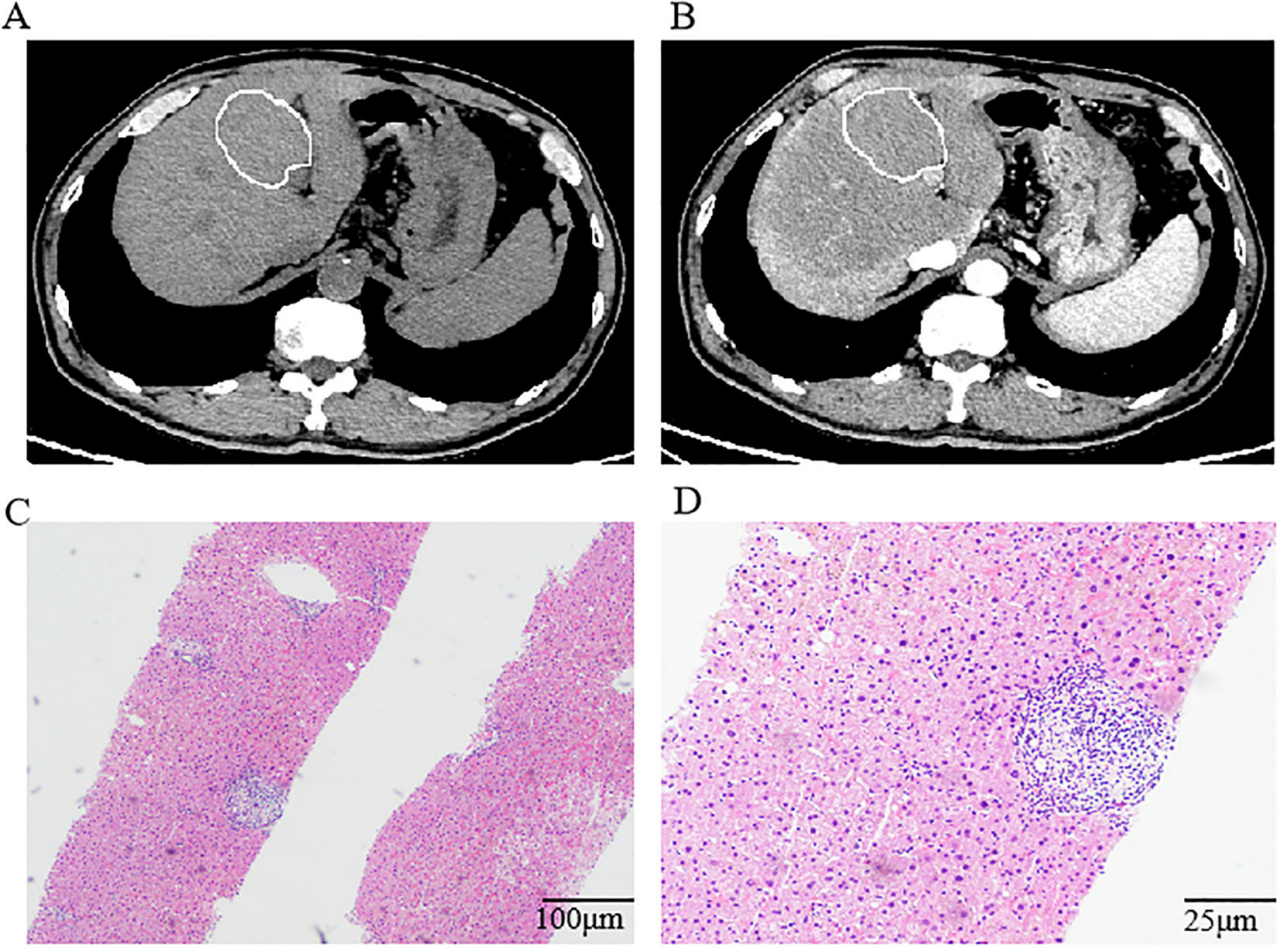
Computed tomography of the abdomen and histological findings of the lesion in the liver. **(A, B)** Computed tomography of the abdomen indicated a low-density lesion in the liver. Histologic examination of the biopsy tissue showing regional watery degeneration of hepatocytes by hematoxylin-eosin stain without a mixed cellular infiltrate of lymphocytes, macrophages, and fibrocytes. Magnification was ×10 **(C)** and ×40 **(D)**.

Following this favorable response, the treatment team recommended transition to tislelizumab monotherapy for maintenance treatment.After completing 17 cycles of maintenance immunotherapy (Month 26 of treatment), chest CT scans indicated stable disease, routine surveillance imaging revealed an incidental hepatic space-occupying lesion(**Figures 2A, B**). To establish definitive diagnosis, ultrasound-guided liver biopsy was performed.

Histopathological examination of the hepatic space-occupying lesion revealed regional watery degeneration of hepatocytes, scattered focal necrosis, mild inflammatory cell infiltration within the hepatic sinuses, fibrous tissue hyperplasia, and a small amount of inflammatory cell infiltration without definite neoplastic changes (**Figures 2C, D**). Immunohistochemical analysis showed GS (-), CD34 (vascular +), β-Catenin (membrane +), CK7 (bile duct +), HSP70 (+), Glypican-3 (-), Ki-67(low expression), Rhodanine (-), and hemosiderin (-). Different combinations of immunohistochemical markers could help to distinguish RHP from other liver space occupying lesions. GS (Glutamine Synthetase) negative staining in RHP helps differentiate it from focal nodular hyperplasia (FNH), which typically shows a characteristic map-like pattern of GS positivity.CD34 positive vascular staining indicates sinusoidal capillarization, a feature often seen in regenerative lesions. This contrasts with normal liver parenchyma, where CD34 staining is limited to portal vessels. β-Catenin membrane-positive staining without nuclear translocation excludes β-catenin-activated hepatocellular adenoma or hepatocellular carcinoma(HCC). This supports the non-neoplastic, regenerative nature of RHP.CK7 (Cytokeratin 7) positive staining in bile ducts confirms the preservation of biliary structures, ruling out cholangiocarcinoma. The absence of CK7 expression in hepatocytes further supports the diagnosis of RHP.HSP70 (Heat Shock Protein 70) positive staining can be seen in both regenerative lesions and HCC. In this case, the absence of other malignant features (e.g., Glypican-3 negativity, low Ki-67) supports a benign, regenerative process. Glypican-3 negative staining is crucial in ruling out HCC, as Glypican-3 is a specific marker for malignant hepatocellular lesions. Ki-67 low proliferative index is consistent with a non-neoplastic, regenerative process. High Ki-67 expression would suggest a malignant lesion.Rhodanine negative staining rules out copper accumulation, which can be seen in Wilson’s disease or chronic cholestatic conditions. Hemosiderin negative staining excludes iron overload, which is associated with hemochromatosis or other iron storage disorders. By integrating these immunohistochemical findings, RHP can be effectively differentiated from other liver lesions, facilitating accurate diagnosis and appropriate clinical management.

The temporal development pattern and histopathological features of the hepatic lesion provided critical evidence for distinguishing tislelizumab-induced regenerative hepatic pseudotumor from other hepatic pathologies. Special investigations, including acid-fast and periodic acid-Schiff (PAS) amylase staining, were negative for atypical fungal and mycobacterial infections. The patient denied any pre-existing granulomatous disease, and a thorough evaluation of viruses, *Mycobacterium tuberculosis*, and autoantibodies yielded negative results. Laboratory tests showed normal levels of AST, ALT, GGT, AKP, bilirubin, and blood calcium. At the time of the initial diagnosis of non-small cell lung cancer (NSCLC), the patient’s liver biochemistry (including ALT, AST, ALP, and bilirubin) and imaging studies (ultrasound and CT scan) were within normal limits, indicating no evidence of pre-existing liver disease. Throughout the six cycles of first-line chemotherapy with albumin-bound paclitaxel and lobaplatin, the patient’s liver function tests remained normal, and no hepatic lesions were detected on imaging. Although chemotherapy drugs are known to cause liver injury, the typical manifestations include steatosis, sinusoidal obstruction syndrome, or nodular regenerative hyperplasia (NRH), rather than regenerative hepatic pseudotumor (RHP). Furthermore, the hepatic lesion developed several months after the completion of chemotherapy, making a direct association with prior chemotherapy unlikely. These findings strongly suggest that the chemotherapy regimen did not contribute to the development of RHP.The hepatic lesion was detected twenty-six months after the initiation of tislelizumab therapy. During this period, the patient did not receive any other hepatotoxic medications or systemic corticosteroids. Additionally, the patient had no history of viral hepatitis, alcohol abuse, or chronic liver disease, further reducing the likelihood of alternative causes for the hepatic lesion.Histopathological examination of the lesion revealed features consistent with RHP, including nodular hyperplasia of hepatocytes with preserved lobular architecture and minimal cytological atypia. These findings are indicative of an immune-mediated process rather than a drug-induced or metabolic etiology.The temporal association between tislelizumab administration and the development of RHP, combined with the absence of alternative etiologies, strongly supports the hypothesis that RHP is an immune-related adverse event (irAE) secondary to tislelizumab therapy.

Based on these findings and the patient’s clinical history, a diagnosis of regenerative hepatic pseudotumor secondary to immunotherapy was made. Oral prednisolone was initiated at 40 mg/day, although it was subsequently discontinued due to multiple skin infections, including oral cold sores. Despite the false tumor-like reaction, re-evaluation of the liver CT showed no significant changes in the mass. Consequently, tislelizumab was discontinued. The patient did not receive any additional antitumor therapy following the RHP diagnosis. CT scans of the chest and abdomen were regularly performed, which showed a persistent partial response to lung cancer. The patient’s prognosis was favorable, with stable clinical condition and no symptoms or liver enzyme abnormalities observed at the time of reporting.

## 3 Patient perspective

Our patient was shocked when he heard the news.While I had experienced significant benefits from Tislelizumab in treating my lung cancer, the discovery of a liver mass filled me with considerable concern. Early liver biopsy and therapeutic consequences was important for the patient to reach a high level of compliance. I found it essential to strictly adhere to my physician’s recommendations, as their guidance provided reassurance and clarity during this challenging time.

## 4 Discussion

Tislelizumab, a new humanized IgG4 PD-1 inhibitor, was approved by China’s National Medical Products Administration (NMPA) in December 2019 for the treatment of classic relapsed or refractory Hodgkin’s lymphoma, locally advanced or metastatic urothelial carcinoma, non-small cell lung cancer, and hepatocarcinoma following at least second-line systemic chemotherapy (11). A number of clinical trials exploring the efficacy of tislelizumab across various indications are underway (12–16). However, adverse reactions associated with tislelizumab remain underreported (17). Common hepatic adverse events observed with tislelizumab therapy include elevated aminotransferase levels, autoimmune hepatitis, and nodular hepatic changes (18). To better understand these adverse events, we searched the available case reports and conducted a literature review of tislelizumab-related adverse events, summarizing our findings in **Table 1**. In the present case, no non-necrotizing granuloma was observed in the liver pathology, and the findings did not meet the diagnostic criteria for nodular reactions. Consequently, the tumor was diagnosed as a RHP.

**Table 1:** Tislelizumab-related adverse events in available case reports.

Adverse reactions	Clinical manifestations	Cycles	Treatment
Kidney and urologic diseases	Ureteritis/cystitis (40)	6	Corticosteroids were the most frequent treatment
	Membranous nephropathy (41)	11	
Blood and lymphatic system disorders	Pancytopenia (42)	2	G-CSF and thrombopoietin (TPO) injection, intravenous (IV) antibiotics platelets and packed red blood cell transfusion; steroids
Immune system	Tumor flare reaction (43)	4	Non-steroidal anti-inflammatory drugs; corticosteroid
Musculoskeletal and connective tissue disorders	Severe myasthenia gravis, myocarditis, rhabdomyolysis (11)	1	Intravenous immunoglobulins (IVIGs) and corticosteroids treatments
Endocrine diseases	Pituitary-adrenal axis dysfunction (44, 45)	3,7	Corresponding hormone replacement
	Thyrotoxicosis (46)	3	Anti-thyroid drugs
Mucosal or cutaneous disease	Pemphigus herpetiformis-type drug reaction (47)	6;	Oral prednisone; glucocorticoid therapy
	Psoriasis (45)	2	
	Steven-Johnson Syndrome/Toxic Epidermal Necrolysis (48, 49)	2	
	Lichen planus pemphigoides (50)	11	
Respiratory diseases	Immune-related pneumonitis (51)	6	Prednisone therapy
Gastrointestinal Diseases	Opportunistic bowel infection (52)	7	Corticosteroid treatment, antiviral drug, antibiotic
Hepatobiliary diseases	Increased levels of liver enzymes (53, 54)	6-14	Discontinuation of checkpoint inhibitor therapy and treatment with immunosuppressive agents
	Autoimmune hepatitis (55)		

A RHP is a non-neoplastic, lumpy lesion that can mimic a tumor on imaging, macroscopic, and histological examinations (8). These lesions are composed entirely of benign reactive parenchyma, distinguishing them from other nodular liver pathologies, such as nodular regenerative hyperplasia, vascular effusion disease, inflammatory pseudotumors, and non-specific changes adjacent to unsampled masses (9). RHP lesions were visible on imaging but were either ill-defined or had indeterminate, making definitive diagnosis reliant on biopsy and histopathological examination (19). Biopsy specimens must adequately sample the lesion to avoid misdiagnosis as non-specific changes adjacent to unsampled mass may obscure the diagnosis (20).

Compared to common hepatic metastases or other immune-related adverse events (irAEs), RHP exhibits distinct pathological characteristics. Histologically, RHP may manifest as focal nodular hyperplasia, inflammatory hepatic adenoma, or segmental atrophy with nodular elastosis (21). Cytologically, individual hepatocytes within the lesion were similar to those outside the lesion. In some cases, hepatocytes were slightly atrophied compared to non-diseased hepatocytes, but there were no differences in morphology and cytological atypia. In contrast, hepatic metastases, particularly from adenocarcinomas, often display glandular or tubular structures with marked cytological atypia, increased mitotic activity, and desmoplastic stroma. Immunohistochemical markers such as CK7, CK20, and CDX2 are commonly used to identify the primary origin of metastatic lesions. Unlike RHP, metastases typically disrupt the normal liver architecture and may exhibit areas of necrosis (22). Furthermore, other irAEs involving the liver, such as immune-mediated hepatitis (irHepatitis), are characterized by diffuse hepatic inflammation, elevated liver enzymes, and histological features of lobular or portal inflammation. irHepatitis is marked by a diffuse inflammatory infiltrate composed predominantly of T lymphocytes and plasma cells, accompanied by hepatocyte injury such as ballooning degeneration, apoptosis, and focal necrosis. Unlike RHP, irHepatitis does not typically form discrete nodular lesions but rather presents as diffuse liver involvement (5, 6). Nodular regenerative hyperplasia (NRH) is another rare pattern of liver injury associated with immune checkpoint inhibitors. NRH is characterized by nodular transformation of hepatocytes without significant fibrosis, often accompanied by vascular changes such as obliterative portal venopathy. Unlike RHP, NRH typically presents as multiple small nodules distributed throughout the liver rather than a single lesion. In summary, RHP can be distinguished from common hepatic metastases, irHepatitis, and NRH based on its unique histological features, cytological characteristics, and lesion distribution (23). Biopsy and excision specimens may identify these radiographically evident lesions, which lack histological features of tumors or pseudotumors but exhibit a unique benign reactive pattern, often in response to abnormal vascular flow (24). There are no data that clearly indicate the causative role of vascular thrombi in these lesions. The natural history of RHP is unclear, but based on available information, some of them stabilize over time, whereas others shrink in subsequent imaging. There was no histological or radiographic evidence of transformation into focal nodular hyperplasia. To date, there is no standard for the diagnosis of immune-associated liver pseudotumors, the potential difficulty in differentiating RHP from other hepatic lesions and the unclear long-term outcomes of RHP, though liver needle biopsy is frequently helpful.

The specific mechanism of RHP remains incompletely understood but may involve excessive immune system activation (1, 2), inflammatory cytokine secretion (5, 6), and vascular changes with perfusion abnormalities (7, 23). In the present study, tislelizumab, a humanized IgG4 anti-PD-1 monoclonal antibody, blocks the PD-1 receptor on T cells, thereby preventing its interaction with PD-L1 expressed on tumor cells and immune-suppressive cells. This inhibition enhances T-cell activation and proliferation, potentially leading to an exaggerated immune response. On the one hand, the liver’s immune microenvironment is highly heterogeneous, and immune checkpoint inhibitors such as tislelizumab may induce localized immune activation rather than a systemic response. This localized activation could result in focal inflammation and subsequent regenerative changes in a specific area of the liver, leading to the formation of a single lesion (25). On the other hand, the liver’s intrinsic heterogeneity in terms of cell composition, metabolic activity, and immune cell distribution may predispose certain regions to localized immune-mediated injury. The Kupffer cells, which have tolerogenic and immune-suppressive functions in homeostasis, may undergo a phenotypic switch and promote tissue remodeling. For instance, variations in Kupffer cell density could explain why lesions develop focally rather than diffusely (26). Moreover, the activation of T cells by PD-1 inhibitors can trigger the release of pro-inflammatory cytokines such as IFN-γ, TNF-α, and IL-6. These cytokines contribute to hepatocyte damage and inflammation, followed by a compensatory regenerative process. The imbalance between tissue injury and repair may result in the formation of pseudotumor-like lesions (27, 28). Additionally, the direct cytotoxic effects of activated T cells on hepatocytes, combined with the liver’s inherent regenerative capacity, may lead to the formation of hyperplastic nodules. This process is distinct from malignant transformation and represents a reactive, non-neoplastic response to immune-mediated injury (29, 30). Furthermore, recent evidence suggests that RHPs are associated with vascular flow abnormalities (31). Localized vascular changes, such as alterations in blood flow or microvascular injury, could contribute to the formation of a single lesion. Tislelizumab has been linked to endothelial cell activation and vascular remodeling, which may induce focal ischemia or perfusion abnormalities in a specific region of the liver (5). These changes can trigger hepatocyte regeneration and the development of RHP.

This case contributes to the growing body of literature on immune-related adverse events (irAEs) in the treatment of malignant tumors. To the best of our knowledge, tislelizumab-induced RHP has never been reported. Notably, liver damage has been recognized as a complication of ICI treatment that manifests as hepatitis with elevated liver enzyme or bilirubin levels. Most of the affected patients have mild disease with no radiological findings or histological features other than symptoms of liver damage. However, in our case, the patient was asymptomatic, with normal laboratory tests were normal, and the only clinical finding was RHP found on liver imaging, which manifested as a single mass with dynamic enhancement. Initially misdiagnosed as metastatic liver disease, the lesion was correctly diagnosed as an RHP based on the histopathological findings. While our diagnosis relied on histopathology, recent advances in circulating tumor DNA (ctDNA) analysis could provide adjunctive molecular insights for RHP cases like ours, particularly when imaging findings are equivocal. In the era of precision oncology, several studies have demonstrated that ctDNA could be used to predict response, monitor response, and study resistance mechanisms to anti-PD-1 therapy (32–34). In the early-stage setting, residual ctDNA after definitive local therapy can be used to identify patients at highest risk of recurrent or metastatic disease (35). There are also important clinical uses of ctDNA in the metastatic setting, which include monitoring tumor evolution (36), evaluating for mechanisms of treatment resistance (37), and deciding when to switch anticancer therapies (38). Longitudinal monitoring of ctDNA has also demonstrated potential to differentiate pseudoprogression from true progression for patients (39). Although ctDNA was not analyzed in our case, future studies could combine histopathology with liquid biopsies to improve diagnostic accuracy.

This is the first reported case of hepatic pseudotumor formation induced by tislelizumab, with efficacy and safety comparable to those of other anti-PD-1 antibodies. Notably, the favorable treatment response observed in this patient, despite the presence of an RHP, supports the hypothesis of a positive association between RHP and favorable treatment outcomes.

In summary, we report a unique case of RHP in a patient with NSCLC treated with tislelizumab and briefly review the clinical features of hepatic pseudotumors associated with ICI treatment. With the increasing global use of the internet, similar cases may become more frequently recognized in the near future. This rare irAE deserves the attention of clinicians, and histopathological evaluation of suspicious lesions that occur after immunotherapy, especially in the case of a mixed response, is critical to ensure appropriate clinical decision-making.

## 5 Conclusion

Reactive hepatic pseudotumor (RHP) is a rare liver manifestation associated with immune checkpoint inhibitors (ICIs) and is not necessarily indicative of disease progression. This case highlights the importance of considering RHP as a potential immune-related adverse event (irAE) in patients receiving ICIs. Early recognition and accurate diagnosis are crucial to avoid unnecessary interventions and ensure appropriate management. Clinicians should maintain a high level of vigilance for hepatic lesions in patients on ICIs, particularly when imaging findings are nonspecific. Biopsy remains the gold standard for diagnosis, and multidisciplinary collaboration is essential for optimizing patient care.

The development of RHP may necessitate temporary or permanent discontinuation of ICIs, depending on the severity of the lesion and the patient’s overall clinical status. This underscores the need for individualized treatment strategies tailored to each patient’s specific circumstances.

This paper has limitations that point to areas for future research. Future studies should focus on the incidence, prevalence, and risk factors of RHP in patients receiving ICIs, particularly tislelizumab. Further research is also needed to elucidate the specific immune mechanisms underlying RHP development, including the roles of T-cell activation, cytokine release, and localized vascular changes. Additionally, predisposing factors for immunotherapy-induced hepatic pseudotumor reactions should be explored, and their impact on non-small cell lung cancer (NSCLC) progression should be evaluated.

The development of standardized diagnostic criteria and management guidelines for RHP and other hepatic irAEs is essential to improve patient outcomes. These guidelines should include recommendations for imaging, biopsy, and treatment strategies. Furthermore, longitudinal studies are needed to assess the long-term outcomes of patients with RHP, including the impact on liver function, tumor response, and overall survival.

## Data Availability

The raw data supporting the conclusions of this article will be made available by the authors, without undue reservation.
