# Correction: Case Report: Regenerative hepatic pseudotumor induced by tislelizumab in a lung cancer patient

**DOI:** 10.3389/fimmu.2025.1654196

**Published:** 2025-07-16

**Authors:** Wenrui Wang, Wei Li, Tianqi Zhang, Zhenjing Jin, Lanlan Yang

**Affiliations:** ^1^ Digestive Diseases Center, Department of Hepatopancreatobiliary Medicine, The Second Hospital, Jilin University, Changchun, China; ^2^ Department of Respiratory and Critical Care Medicine, The Second Hospital of Jilin University, Changchun, Jilin, China; ^3^ Department of Radiology, The Second Hospital of Jilin University, Changchun, China

**Keywords:** immune checkpoint inhibitors, immune-related adverse events, non-small cell lung cancer, regenerative hepatic pseudotumor, tislelizumab, hepatic immune toxicity, PD-1 inhibitors, immunotherapy complications

In the published article, there was an error in the **Funding** statement. The funder “Natural Science Foundation of Jilin Province (YDZJ202501ZYTS814 and YDZJ202401255ZYTS) to Wenrui Wang” was erroneously omitted.

The correct **Funding** statement appears below.

“The author(s) declare that financial support was received for the research and/or publication of this article. Natural Science Foundation of Jilin Province (YDZJ202501ZYTS814 and YDZJ202401255ZYT) provided funding to author Wenrui Wang.”

The authors apologize for this error and state that this does not change the scientific conclusions of the article in any way. The original article has been updated.

